# Clinical and radiographic evaluation of the efficacy of vital- os bone cement in stabilizing an autogenous monocortical bone graft in the repair of oroantral fistula (a comparative study)

**DOI:** 10.1007/s44445-025-00037-8

**Published:** 2025-07-15

**Authors:** Aliaa A. Habib, Mahitab M. Soliman, Mervat M. Khalil

**Affiliations:** Oral and Maxillofacial Surgery Department, Faculty of Dentistry, Pharos University in Alexandria, 22 Canal Suiz Street, Moharm Bek, Alexandria, 21515 Egypt

**Keywords:** Oroantral communication, Vital-Os bone cement, Computerized tomography

## Abstract

One clinical complication oral surgeons encounter is oroantral communication (OAC) with progressive formation of oroantral fistula (OAF). Among bone grafting materials, autogenous bone is still considered the gold standard in grafting oral and maxillofacial regions. To evaluate clinically and radiographically the efficacy of Calcium phosphate (Vital-Os) bone cement in stabilizing an autogenous bone graft in repairing an oroantral fistula. The study recruited 20 patients with an oroantral fistula ranging from 4–10 mm diameter. Ten patients were treated by grafting the defect with bone graft harvested from the chin area and closure of oroantral by buccal advancement flap and Vital-Os Bone cement (Study group), and ten patients were treated without Vital-Os Bone cement (Control group). All patients came for postoperative follow-up at the 1st, 3rd, and 6th months. Visual Analogue Scale (VAS) in the study group was significantly lower than the control group one week and one month postoperatively (*p* = .007, .004; respectively). Failure of the oroantral fistula closure was significantly lower in the study group (*p* = .025). The closure of an oroantral fistula is recommended through the use of an autogenous bone graft harvested from the chin region, supplemented by the application of injectable VitalOs bone cement to secure the graft at the recipient site.

**Supplementary Information:**

The online version contains supplementary material available at 10.1007/s44445-025-00037-8.

## Introduction

One of the clinical complications encountered by oral surgeons is oroantral communication (OAC) with the progressive formation of an oroantral fistula (OAF) (Ogunsalu 2005).

Many techniques have been proposed to seal the socket from the oral environment. Soft tissue closure using different flap designs that cover these defects has been reported, such as buccal flap, palatal rotation flap, tongue flap, and buccal pad of fat (Kraut 2000).

Different bone graft materials are available and have been used to close oroantral communications. Bone grafts are divided into four categories: autografts, allografts, alloplasts, and xenografts (Scattarella et al. 2010).

Autogenous bone grafts are generally obtained from extraoral sites or intraoral sites. The extraoral grafts can be easily obtained from the ilium, the rib, and the calvarium, but each site has morbidity associated with complex surgical procedures (Finkemeier 2002).

Whether put in the form of particles or blocks, the risk of displacement of the graft material into the antrum still presented a hazard (Schwartz-Arad 2007). Thus, a guided tissue regeneration membrane combined with a bone graft was suggested to close an oroantral fistula (Waldrop 1993).

Since the appearance of bone cement in 1980, it has been used to fix bone fractures, stabilize implants, and fill bony defects in the oral cavity (Lim et al. 2010).

Bone cement is considered one of the alloplastic bone graft materials; it is in the form of granules or blocks made up of β-TCP, hydroxyapatite, or a mix of both (Flautre et al. 2003).

Calcium phosphate (VitalOs) bone cement was used for bone regeneration in dental osseous defects, treatment of mobile teeth in periodontal defects, and maxillofacial fractures to be pressed into all small openings and voids in the spongy skeleton to fill all hollows on uneven fracture surfaces (Lu et al. 2002).

In an attempt to avoid voids around the bone graft used to prevent displacement, the addition of bone cement around the graft will be investigated in this study.

## Materials and methods

The study recruited 20 patients with an oroantral fistula ranging from 4–10 mm diameter. Ten patients were treated by grafting the defect with the closure of oroantral communication by buccal advancement flap and Vital-Os Bone cement(Study group). Ten patients were treated by grafting the defect with the closure of oroantral communication by buccal advancement flap (Control group).To stabilize the bone graft in oroantral bone defect, a monocortical bone graft was harvested from the chin area by trephine bur.

### Criteria of selection

#### Inclusion criteria

- Patients in this clinical study suffer from an oroantral fistula ranging from 4–10 mm in diameter.

#### Exclusion criteria

- Patients suffering from an oroantral fistula more than 10 mm in diameter.
- Patients with degeneration in the sinus membrane.
- Patient with a history of systemic illness.

### Materials

#### Instrument

- Surgical autoclavable graduated trephine bur with different sizes (Tut company**)**^1^^*^
- Contra-angle low-speed handpiece with micro motor.
- A caliper was used to measure the bone defect size.

#### VitalOs bone cement (Webb 2007)

It is a resorbable calcium phosphate-based hydraulic cement in injectable form.

### Preoperative phase

#### Clinical examination

- **Extra oral examination:** Upon visual inspection, all patients were assessed for the presence or absence of swelling in the area associated with the oroantral fistula (OAF). Through palpation, the examination focused on detecting tenderness in the cheek region and identifying any palpable lymph nodes.
- **Intraoral examination:** Upon examination, the following observations were made: the location of the Oroantral Fistula (OAF) was identified, the presence or absence of polyps was noted, and the presence of purulent discharge from the fistula was assessed. Through palpation, the area was evaluated for tenderness in the region of the OAF.

#### Radiographic examination

Axial, coronal, three-dimensional dental computed tomography to measure the diameter of OAF.

#### Preoperative preparation of the patient

- A culture and sensitivity test determined the specific antibiotics.
- Using sterile normal saline, antral lavage of the sinus was done daily for 2 weeks.
- Supra and subgingival scaling and root planning were done.

### Operative phase

#### Anesthesia

All surgical procedures were carried out under local anesthesia. The operation was performed in the Oral and Maxillofacial Surgery Department, Faculty of Dentistry, Pharos University in Alexandria.

#### Surgical procedures

A circular excision around the orifice of the fistula to remove the entire epithelialized tract (Decoring) was made using a No.11 Bard Parker Blade. A buccal advanced mucoperiosteal flap was done: Using No.15 Bard Parker Blade, two vertical divergent incisions were made through the buccal mucoperiosteum and extended superiorly on each side of OAF. The full-thickness flap was then elevated. A small curette and bone file were used to remove all the necrotic bone fragments from the socket, expose all bony margins of the defect, and allow adequate sinus examination. Remove any remaining roots or foreign bodies. The irregular margins of the bony defect were trimmed to the smallest possible rounded shape. The rounded bony defect was measured by a caliper.

Sinus irrigation was done several times through the defect using sterile normal saline until a clear washout was obtained. A releasing incision was made in the periosteum on the underside of the flap at the depth of the mucobuccal fold to allow flap mobilization and suturing without tension. The recipient site was covered temporarily with gauze until bone graft harvesting was done.

The mucoperiosteal flap was incisioned from the lower right canine to the lower left canine and then reflected to expose the symphysial area. A monocortical block graft (corticocancellous) was harvested using a micromotor with low-speed contra-angle and a trephine bur under a cooling system with an inner diameter matching the size of the bony defect (whose determined by caliper and three-dimensional CT measurements).

The graft was dislodged from the bone using osteotome and then transferred to the recipient site. The graft was press-fit into the defect by friction. VitalOs bone cement was injected around the bone graft to fill the voids between the graft and the bone defect (for the study group only). The injected cement paste can be shaped within the first 4 minutes after the cement is left untouchable for the remaining 7 minutes needed for setting. In contrast, the total hardening time is less than 13 minutes.

The buccal advancement flap was repositioned after hardening the bone cement by using 000 Vicrel suture material. The lower mucoperiosteal flap was repositioned and sutured with a horizontal mattress and interrupted sutures using 000 Vicrel suture material.

### Postoperative phase

#### Immediate postoperative phase

- Patients are advised to consume a soft diet during the first week following surgery and to avoid foods that are hot, salty, or acidic. It is also recommended to prevent any negative or positive pressure within the sinus, which can be caused by actions such as nose blowing, sneezing with a closed mouth, and smoking.
- All patients were administered postoperative antibiotics, selected based on culture and sensitivity tests, for one week following surgery. Additionally, anti-inflammatory medication, specifically Brufen 400 mg, was prescribed to be taken three times daily for one week. Nasal decongestants, such as Otrivin 1% nasal drops, were recommended to be used three times daily for two weeks to alleviate nasal obstruction and promote drainage.

#### Delayed postoperative phase (follow-up)

##### **Pain**

Visual Analogue Scale (VAS) was used to analyze pain. A zero to ten (0-1= None, 2-4= Mild, 5-7= Moderate, 8-10= Severe) scale. All patients were instructed to come for postoperative follow-up after 2 days, one week, and one month to evaluate pain (Seymour et al. 1985).

##### **Wound healing**

For assessment of wound healing, the intraoral incision was regularly assessed and monitored for any indications of dehiscence, infection, or inflammation during the postoperative period by Landry's healing index (Pippi RJIjoms 2017).

##### **Computerized tomography**

**Fig. 1:**
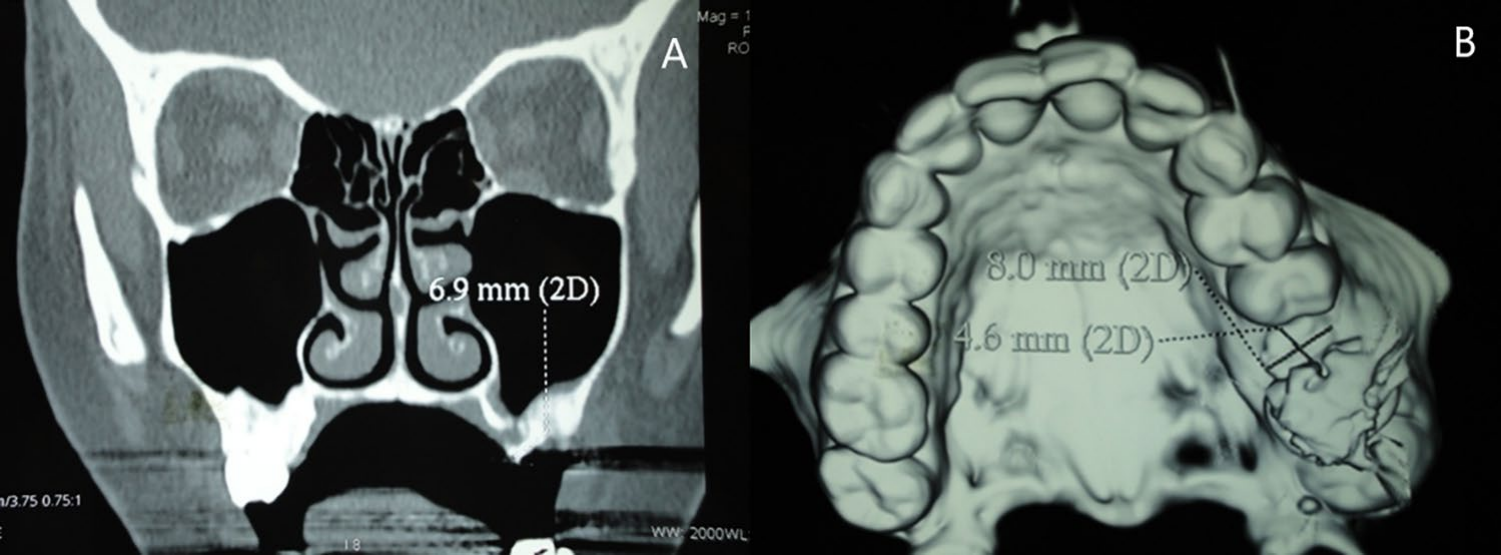
(**A**) Coronal CT showing the closure of the left-sided oroantral fistula without any infection or opacity of the maxillary sinus at the end of the sixth month postoperatively **(B)** Three-dimensional CT showing the closure of the cortical bone defect at the base of the extracted socket of the left first molar (site of the oroantral fistula) at the end of the sixth month postoperatively (Study group)

**Fig. 2:**
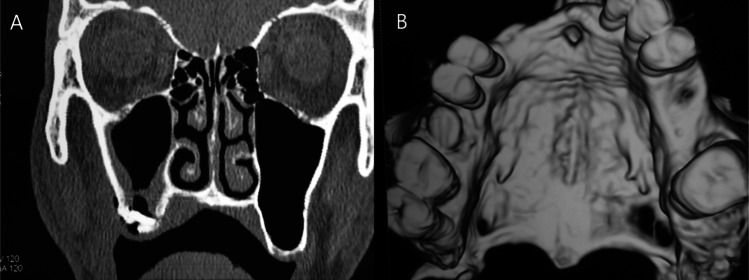
(**A**) Coronal CT showing the closure of the right-sided oroantral fistula without any infection or opacity of the maxillary sinus at the end of the sixth month postoperatively **(B)** Three-dimensional computerized tomography showing the closure of the cortical bone defect at the base of the extracted socket of the left first molar (site of the oroantral fistula) at the end of the sixth month postoperatively. (Control group)

Continuous coronal scans of the maxillary sinus to determine the sinus condition and the closure of bony defect by the monocortical bone graft, computerized tomography was done at the end of the 6^th^ month postoperatively. (Fig. 1 A, B) (Fig. 2 A, B)

##### Post-hoc power analysis (sample size justification)

The power achieved by sample size of 10 patients per group (number of groups = 2) for t test (Means: Differences between two independent means (two groups)) based on comparison of VAS one week and one month postoperative resulted in two-tails standardized effect size (d) of 1.506, and 1.566; respectively) and a power of 88.97, 91.15% (Montgomery 2020).

##### **Statistical methodology**

Shapiro–Wilk test of normality was used to assess the distribution of the quantitative variables (Shapiro and Wilk 1965; Shapiro 2015). Most continuous variables were not normally distributed, so non-parametric statistics were *adopted* (Field 2013*).* Mann–Whitney U test (Mann and Whitney 1947) was used for between group comparison. Z-test for independent proportions (Sprinthall 2013) is used to compare closure of the oroantral fistula 6 months postoperatively. The sample size justification was carried out at 80%. An alpha level was set to 5% with a significance level of 95%. Statistical significance was tested at *p*-value < 0.05 (Curran-Everett DJAipe. 2020).

All enrolled participants (n = 20) completed the study protocol. There were no dropouts or losses to follow-up.

## Results

The median age of the study group was 20.50 years (60% were males), and for the control group was 23.50 years (60% were males), with no statistically significant difference between the two studied groups regarding age and sex (*p* = 0.183, *p* = 1.000; respectively).

VAS was not significantly different between the two studied groups two days postoperatively (*p* = 0.104). However, the study group showed a statistically significantly lower VAS compared with the control group one week and one month postoperatively (*p* = 0.007, 0.004; respectively) (Table 1).

**Table 1:** VAS of the studied groups at different time intervals

VAS	Group		Test of significance *p*-value
	Study Group (n = 10)	Control Group (n = 10)	
Two days postoperative			
- Min. – Max	5.00–7.00	5.00–8.00	
- Mean ± SD	6.00 ± 0.82	6.70 ± 0.95	Z_(MW)_ = 1.627
- Median	6.00	7.00	*p* =.104 NS
- 25th Percentile 25th – 75th Percentile	5.00–7.00	6.00–7.00	
One week postoperative			
- Min. – Max	1.00–3.00	2.00–5.00	
- Mean ± SD	2.10 ± 0.74	3.40 ± 0.97	Z_(MW)_ = 2.714
- Median	2.00	3.50	*p* =.007*
- 25th Percentile 25th – 75th Percentile	2.00–3.00	3.00–4.00	
One month postoperative			
- Min. – Max	0.00–1.00	1.00–3.00	
- Mean ± SD	0.50 ± 0.53	1.60 ± 0.84	Z_(MW)_ = 2.911
- Median	0.50	1.00	*p* =.004*
- 25th Percentile 25th – 75th Percentile	0.00–1.00	1.00–2.00	

*n* Number of patients, *Min–Max* Minimum – Maximum, *SD* Standard Deviation, *CI* Confidence interval, *MW* Z Test of Mann–Whitney Test, * Statistically significant (*p* <.05), *NS* Statistically not significant (*p* ≥.05)

Healing of Soft Tissue Index (HSTI) was statistically significantly higher in the study group at two days postoperative (*p* = 0.002). Higher level also in the study group, with a difference that tends to be near significant level (~ 0.05) at one week, one month postoperatively (*p* = 0.068, *p* = 0.089; respectively). There was no statistical significance at three months postoperatively (*p* = 0.170) (Table 2).

**Table 2:** Healing of soft tissue index of the studied groups at different time intervals

Healing of soft tissue index	Group		Test of significance *p*-value
	Study Group (n = 10)	Control Group (n = 10)	
Two days postoperative			
- Min. – Max	3.00–4.00	2.00–3.00	
- Mean ± SD	3.40 ± 0.52	2.40 ± 0.52	Z_(MW)_ = 3.130
- Median	3.00	2.00	*p* =.002*
- 25th Percentile – 75th Percentile	3.00–4.00	2.00–3.00	
One week postoperative			
- Min. – Max	3.00–5.00	2.00–4.00	
- Mean ± SD	3.70 ± 0.67	3.00 ± 0.82	Z_(MW)_ = 1.823
- Median	4.00	3.00	*p* =.068 NS
- 25th Percentile – 75th Percentile	3.00–4.00	2.00–4.00	
One month postoperative			
- Min. – Max	4.00–5.00	3.00–5.00	
- Mean ± SD	4.50 ± 0.53	4.00 ± 0.67	Z_(MW)_ = 1.699
- Median	4.50	4.00	*p* =.089 NS
- 25th Percentile – 75th Percentile	4.00–5.00	4.00–4.00	
Three months postoperative			
- Min. – Max	4.00–5.00	4.00–5.00	
- Mean ± SD	4.80 ± 0.42	4.50 ± 0.53	Z_(MW)_ = 1.371
- Median	5.00	4.50	*p* =.170 NS
- 25th Percentile – 75th Percentile	5.00–5.00	4.00–5.00	

*n* Number of patients, *Min–Max* Minimum – Maximum, *SD* Standard Deviation, *CI* Confidence interval, *MW* Z Test of Mann–Whitney Test, * Statistically significant (*p* <.05), *NS* Statistically not significant (*p* ≥.05)

Six months postoperative, radiographic evaluation of closure of the oroantral fistula with an autogenous bone graft was statistically significantly higher (100%) in the VitalOs bone cement group compared with 60% in the control group (*p* = 0.025).

## Discussion

OAF is a pathological condition characterized by the existence of communication between the oral cavity and the maxillary sinus as a result of the loss of the soft and hard tissue that normally separates both compartments (Hirata et al. 2001).

Defects that are larger than 5 mm in diameter or those that present for more than 3 weeks fail to heal spontaneously and require surgical intervention by creating a barrier between the oral mucosa and maxilla antral mucosa (Nagaraj et al. 2018).

The assessment criteria for the choice of techniques are guided by: (a) size and type of defect, (b) presence or absence of sinus infection, (c) minimal donor site morbidity to the patient, and (d) prosthetic considerations and experience of the operating surgeon (Parvini et al. 2019).

Proctor (Proctor 1969) claimed that the insertion of autogenous bone grafts into the bony fistula supported the buccal advancement flap. In our study, we used monocortical bone grafts harvested from the mandibular symphysis. The use of autogenous bone grafts was based on the fact that they provided the most rapid and predictable formation of bone regarding bone quality and quantity (Nkenke et al. 2001).

The monocortical grafts require a short healing period, exhibit minimal resorption, and maintain their dense quality (Schwartz-Arad 2007). While bicortical grafts were avoided since they require taking a portion of the inferior border, that might decrease the structural support of the mandible (Smith and Schaberg 1982).

Haas et al. (Haas et al. 2003). used an autogenous monocortical bone graft to fill the oroantral bony defect by press-fit. They reported that some of their cases needed additional internal fixation with miniplates or screws to avoid mobility of the bone graft. Thus, in a trial to prevent graft displacement and omit the need for internal fixation.

Eduardo et al.(Araújo et al. 2019) used a system 1.5 mm with an"L"plate and four bolts to fix the grafting bone block from the anterior wall of the left maxillary sinus during a surgical procedure for the oroantral fistula.

Verma (Verma 2022) used the technique of three-tier management with septal cartilage placement from the antral side after elevating the mucosal lining and fat, stitched to the advanced buccal flap at the medial end. Septal cartilage is being used in otolaryngological practice with advantages over other materials in many reconstructive procedures. Autologous cartilage is biocompatible, non-absorbable, manipulable, durable, non-carcinogenic, easily available, resistant to infection, and cost-effective.

Kumar (Kumar and Singh NJJoM, Surgery O. 2021) used a modified inverted periosteal flap technique for OAF closure. It preserves vestibular depth, maintains keratinized gingiva width, providing true tension free closure with easier access and manipulation of the periosteal flap.

This study was conducted on twenty patients suffering from an oroantral fistula on top of tooth extraction. According to the literature, satisfactory soft tissue coverage was recommended for the closure of an oroantral fistula.

The buccal advancement flap was used for all patients since its viability is very good, owing to its broad base and thickness. In addition, this method is also applicable to the closure of a very large fistula (Schwartz-Arad and Levin 2005). On exposing the site of the oroantral fistula, clinical measurements of the bone defects coincided with the measures obtained via the preoperative three-dimensional CT.

In the cases of the study group, bone grafts whose size perfectly coincided with the defect size and press fit were obtained. The use of VitalOs bone cement secures the graft at the recipient site. This was believed to maintain volume stability and speed up revascularization by bringing the graft closer to the surrounding blood vessels. The ingrowth of blood vessels into the cortical bone was promoted by peripheral osteoclastic resorption and vascular infiltration of Volkmann's and Haversian canals (Yilmaz et al 2003).

None of the study cases showed flap dehiscence, which could be attributed to the advantage offered by the buccal advancement flap, the stabilization of the graft, and the use of VitalOs bone cement.

According to the review of scientific studies on the present components of VitalOs bone cement, no carcinogenic or mutagenic effects have been observed, nor has any allergenic or sensitization impact been noted (Cuisinier et al. 2004).

In our study, the follow-up X-ray at the end of the sixth month showed partial graft resorption. This was expected, as Phillips's findings indicate that bone grafts transplanted to hard tissue defects undergo cellular regeneration followed by remodeling, which suggests that bone metabolism is associated with cycles of active bone resorption and new bone formation (Silva et al. 2006).

## Conclusion

The closure of an oroantral fistula is recommended through the use of an autogenous bone graft harvested from the chin region, supplemented by the application of injectable VitalOs bone cement to secure the graft at the recipient site.

## Supplementary Information

Below is the link to the electronic supplementary material.

Supplementary file1 (PNG 420 KB)

Supplementary file1 (TIF 2578 KB)

Supplementary file2 (PNG 1.09 MB)

Supplementary file2 (TIF 2810 KB)

## Data Availability

The authors confirm that the data supporting the findings of this study are available within the article and its supplementary materials.

## Footnotes

1 Egyptian Co. for Dental Implants, Egypt.

## References

[CR1] Ogunsalu CJWIMJ (2005) A new surgical management for oroantral communication. 54(4):261–3. 10.1590/s0043-31442005000400011 16312195

[CR2] RA Kraut RA, Smith RVJAoto, America mscoN (2000) Team approach for closure of oroantral and oronasal fistulae. 8(1):55-75. 11212387

[CR3] Scattarella A, Ballini A, Grassi FR, Carbonara A, Ciccolella F, Dituri A, et al (2010) Treatment of oroantral fistula with autologous bone graft and application of a non-reabsorbable membrane.7(5):267. 10.7150/ijms.7.267 PMC2920572 20714437

[CR4] Finkemeier CGJJ (2002) Bone-grafting and bone-graft substitutes. 84(3):454-64. 10.2106/00004623-200203000-00020 11886919

[CR5] Waldrop TC, Semba SEJJop (1993) Closure of oroantral communication using guided tissue regeneration and an absorbable gelatin membrane. 64(11):1061-6 10.1902/jop.1993.64.11.1061 8295091

[CR6] Lim HC, Sohn JY, Park JC, Um YJ, Jung UW, Kim CS, et al (2010) Osteoconductive effects of calcium phosphate glass cement grafts in rabbit calvarial defects. 95(1):47-52. 10.1002/jbm.b.31681 20665684

[CR7] Flautre B, Lemaitre J, Maynou C, Van Landuyt P, Hardouin PJJoBMRPAAOJoTSfB, The Japanese Society for Biomaterials, Biomaterials TASf, et al (2003) Influence of polymeric additives on the biological properties of brushite cements: an experimental study in rabbit. 66(2):214–23. 10.1002/jbm.a.10539 12888990

[CR8] Lu J, Descamps M, Dejou J, Koubi G, Hardouin P, Lemaitre J, et al (2002) The biodegradation mechanism of calcium phosphate biomaterials in bone. 63(4):408-12 10.1002/jbm.10259 12115748

[CR9] Webb J, Spencer RJTJoB (2007) Volume JSB. The role of polymethylmethacrylate bone cement in modern orthopaedic surgery. 89(7):851-7 10.1302/0301-620X.89B7.19148 17673574

[CR10] Seymour RA, Simpson JM, Charlton JE, Phillips MEJP (1985) An evaluation of length and end-phrase of visual analogue scales in dental pain. 21(2):177-85 10.1016/0304-3959(85)90287-8 3982841

[CR11] Pippi RJIjoms (2017) Post-surgical clinical monitoring of soft tissue wound healing in periodontal and implant surgery. 14(8):721. 10.7150/ijms.19727 PMC5562125 28824306

[CR12] Montgomery DC (2020) Introduction to statistical quality control: John wiley & sons.

[CR13] Shapiro SS, Wilk MB (1965) An analysis of variance test for normality (complete samples). Biometrika 52(3/4):591–611

[CR14] Shapiro S (2015) The Shapiro-Wilk and related test for normality. Statistics (Ber).

[CR15] Field A (2013) Discovering Statistics Using IBM SPSS Statistics, 4th edn. SAGE Publications Ltd, London, California, New Delhi

[CR16] Mann HB, Whitney DR (1947) On a test of whether one of two random variables is stochastically larger than the other. The annals of mathematical statistics. 1947:50–60.

[CR17] Sprinthall RC (2013) Basic Statistical Analysis: Pearson New International Edition: Pearson Education, Limited.

[CR18] Curran-Everett DJAIPE (2020) Evolution in statistics: P values, statistical significance, kayaks, and walking trees. American Physiological Society Bethesda, MD; p. 221–4. 10.1152/advan.00054.2020 32412384

[CR19] Hirata Y, Kino K, Nagaoka S, Miyamoto R, Yoshimasu H, Amagasa TJKGzTJotSS, Japan (2001) A clinical investigation of oro-maxillary sinus-perforation due to tooth extraction. 68(3):249-53. 10.5357/koubyou.68.249 11605197

[CR20] Nagaraj T, Sahu P, Saxena S, Nigam H, Biswas AJJOM (2018) Radiology, Pathology, Surgery. Oroantral fistula: a case report and review of literature. 5(4):8.

[CR21] Parvini P, Obreja K, Begic A, Schwarz F, Becker J, Sader R, et al (2019) Decision-making in closure of oroantral communication and fistula. 5:1-11 10.1186/s40729-019-0165-7 PMC6441669 30931487

[CR22] Proctor BJTL (1969) Bone graft closure of large or persistent oromaxillary fistula. 79(5):822-6. 10.1288/00005537-196905000-00005 4890238

[CR23] Nkenke E, Schultze‐Mosgau S, Kloss F, Neukam F, Radespiel‐Tröger MJCoir (2001) Morbidity of harvesting of chin grafts: a prospective study. 12(5):495-502 10.1034/j.1600-0501.2001.120510.x 11564110

[CR24] Schwartz-Arad D, Levin LJId (2007) Multitier technique for bone augmentation using intraoral autogenous bone blocks. 16(1):5-12 10.1097/ID.0b013e3180327595 17356367

[CR25] Smith TS, Schaberg SJ, Collins JTJJoO, Surgery M (1982) Rapair of a palatal defect using a dorsal pedicle tongue flap. 40(10):670-3 10.1016/0278-2391(82)90120-3 6956692

[CR26] Haas R, Watzak G, Baron M, Tepper G, Mailath G, Watzek GJOS, Oral Medicine, Oral Pathology, Oral Radiology, et al (2003) A preliminary study of monocortical bone grafts for oroantral fistula closure. 96(3):263–6. 10.1016/s1079-2104(03)00375-5 12973280

[CR27] De Araújo JCWP, Neto JMB, de Araújo Júnior JL, Ribeiro ED, Rocha JFJAOHI (2019) Use of autogenous bone graft of the anterior wall of the maxillary sinus in the management of oroantral fistula. 8(9).

[CR28] Verma RR, Verma RJIJoO, Head, Surgery N (2022) Oro-antral fistulas and their management: our experience. 74(Suppl 2):1576-83 10.1007/s12070-021-02739-x PMC9702377 36452794

[CR29] Kumar A, Singh NJJoM, Surgery O (2021) Modified inverted Periosteal Flap Versus Buccal Advancement flap technique for Oroantral Fistula Repair: a comparison. 1–6.

[CR30] Schwartz-Arad D, Levin L, Sigal LJId (2005) Surgical success of intraoral autogenous block onlay bone grafting for alveolar ridge augmentation. 14(2):131-8. 10.1097/01.id.0000165031.33190.0d 15968184

[CR31] Yilmaz T, Suslu AE, Gursel BJAjoo (2003) Treatment of oroantral fistula: experience with 27 cases. 24(4):221–3. 10.1016/s0196-0709(03)00027-9 12884211

[CR32] Cuisinier F, Schaaf J, Van Landuyt P, Roth D, Lemaitre J, Tenenbaum HJJoAB et al (2004) Immediate implant placement using injectable calcium phosphate hydraulic cement in dogs. 2(2):88–95. 20803442

[CR33] Silva FMS, Cortez ALV, Moreira RWF, Mazzonetto RJId (2006) Complications of intraoral donor site for bone grafting prior to implant placement. 15(4):420-6 10.1097/01.id.0000246225.51298.67 17172961

