# Supplementary figures and images for: Clinical and radiographic evaluation of the efficacy of vital- os bone cement in stabilizing an autogenous monocortical bone graft in the repair of oroantral fistula (a comparative study)
Source: Saudi Dent J. 2025 Jul 15;37(4-6):30. doi: 10.1007/s44445-025-00037-8 (PMC12263495; doi:10.1007/s44445-025-00037-8)

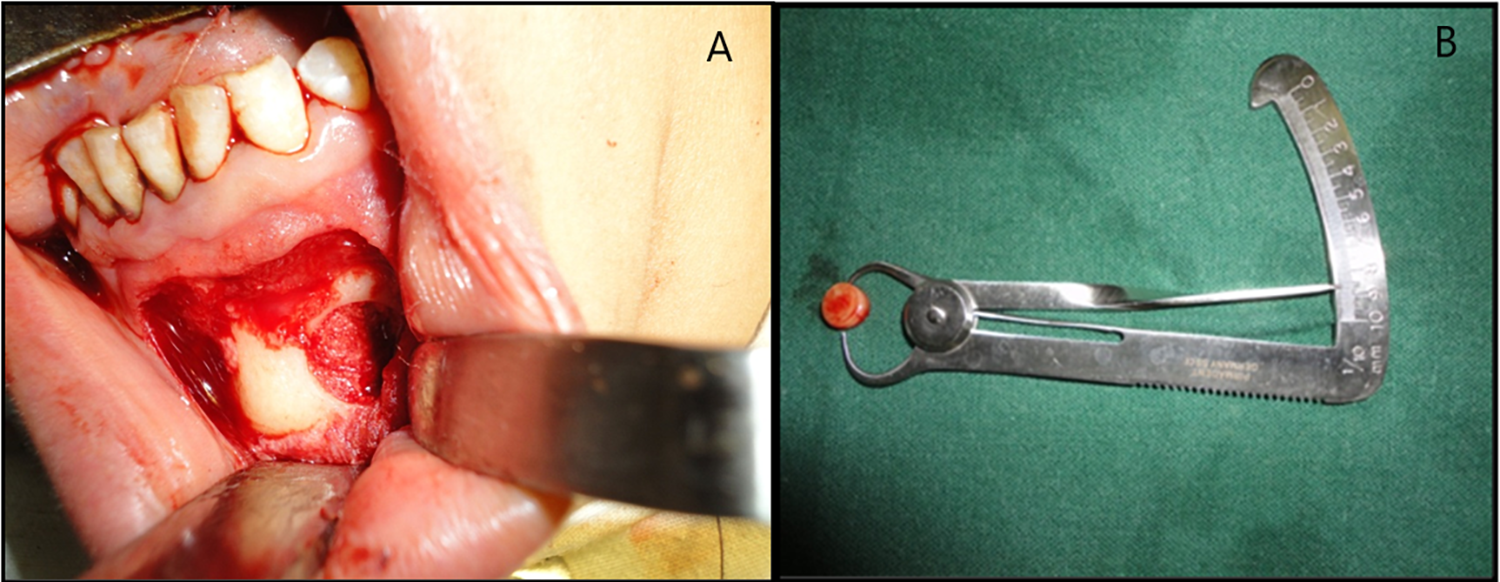

Supplement: Supplementary file 1 — (PNG 420 KB) [file 44445_2025_37_Fig3_ESM.png]

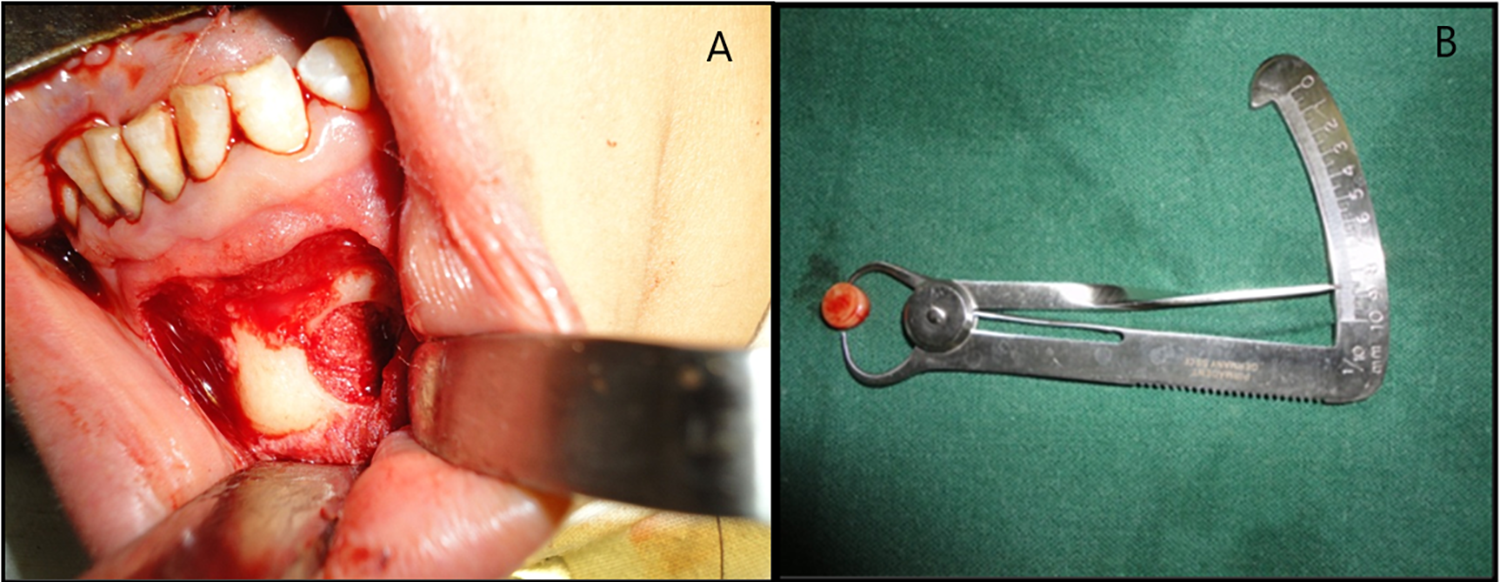

Supplement: Supplementary file 2 — Supplementary file1 (TIF 2578 KB) [file 44445_2025_37_MOESM1_ESM.tif]

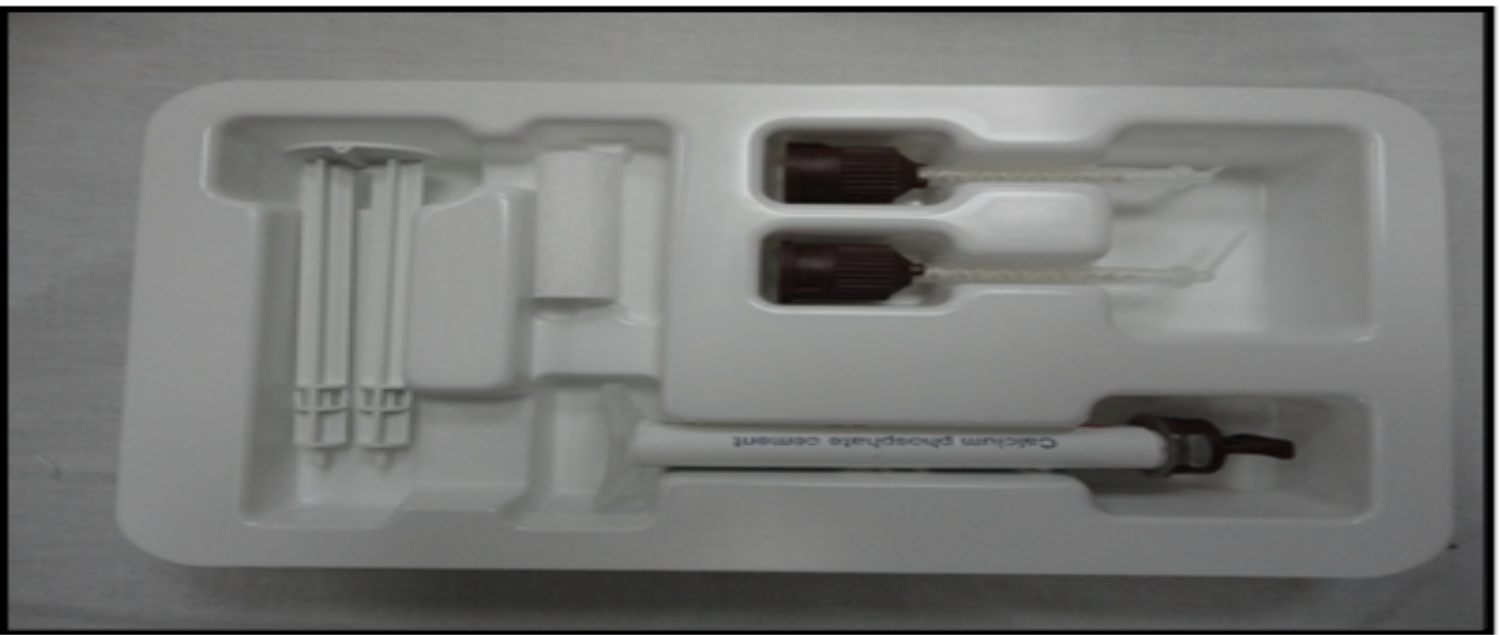

Supplement: Supplementary file 3 — (PNG 1.09 MB) [file 44445_2025_37_Fig4_ESM.png]

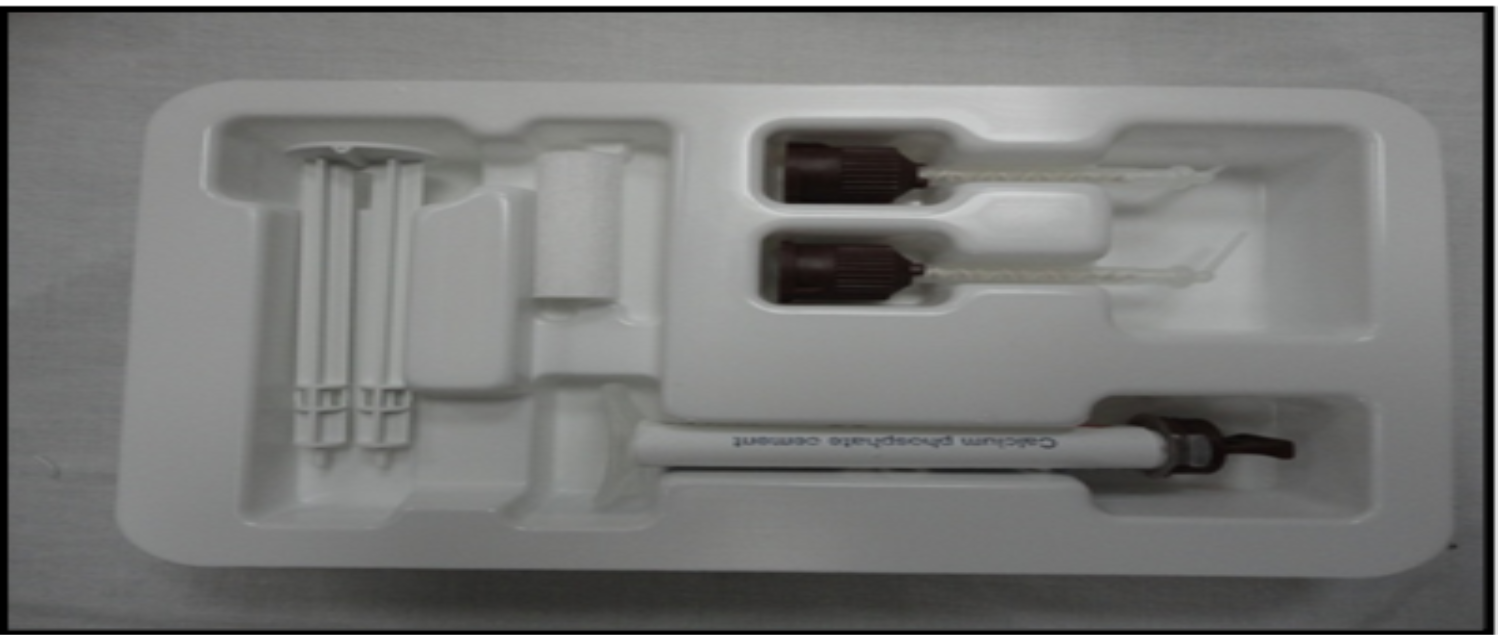

Supplement: Supplementary file 4 — Supplementary file2 (TIF 2810 KB) [file 44445_2025_37_MOESM2_ESM.tif]
